# The Association Between Soil Sampling and Bait Traps in Wireworm Monitoring: A Methodological and Statistical Approach

**DOI:** 10.3390/insects17040419

**Published:** 2026-04-15

**Authors:** Lorenzo Furlan, Giancarlo Bourlot, Annalisa Turchi, Valerio Snichelotto, Maddalena Cappello Fusaro, Stefano Bona

**Affiliations:** 1 Veneto Agricoltura, 35020 Legnaro, Italy; 2 Department of Agriculture and Food, Settore Attuazione Programmi Agroambientali e per l’Agricoltura Biologica Della Regione Piemonte, 10127 Turin, Italy; giancarlo.bourlot@regione.piemonte.it; 3 Independent Researcher, 10051 Avigliana, Italy; lisolo@libero.it; 4 Independent Researcher, 45100 Rovigo, Italy; valeriosn7@gmail.com; 5 Department of Agronomy, Food, Natural Resources, Animals and Environment, University of Padova, 35020 Legnaro, Italy; maddalena.cappellofusaro@unipd.it (M.C.F.); stefano.bona@unipd.it (S.B.)

**Keywords:** *Agriotes*, *Agriotes brevis*, *Agriotes sordidus*, *Agriotes ustulatus*, IPM, damage thresholds

## Abstract

The key to implementing IPM of wireworms effectively is to associate feasible, reliable and affordable sampling methods with well-defined damage thresholds. As wireworms live underground, they cannot be observed directly, thus estimating population levels can be challenging. Soil sampling to ascertain larval density is very time-consuming, and although the use of bait traps is much more time-effective, it is unclear how wireworm numbers in bait traps are associated with wireworm density. The work described herein was conducted between 1993 and 1999 in two regions of Northern Italy: Veneto and Piedmont. The experimental protocol involved placing soil bait traps in a 15–30 m × 10 m grid in selected cultivated fields and taking a soil sample 3 m from the location of each bait trap. The number of monitoring points ranged from 12 to 48 per site. Both trap contents and soil cores were put in funnels to dry out, forcing the wireworms to move and fall into a vial, according to the Berlese method. A moderate association was found between the number of wireworms (*Agriotes brevis*, *A. sordidus* and *A. ustulatus*) caught by the bait traps and by soil sampling, indicating a potential for reciprocal estimation between methods. In other words, the number of bait-trap catches can be estimated by soil sampling (e.g., when bait traps cannot be used due to low temperatures or when growing plants cover a field) and vice versa. The potential of bait traps for catching wireworms was shown to be 5 to 25 times higher than the potential of soil sampling. The threshold values for soil samples, which were derived from the original bait-trap values, range between 15 and 20 larvae/m^2^.

## Simple Summary

Wireworms are soil-dwelling insect larvae that may cause damage to crops such as maize, potatoes and sunflower. Environment-harmful insecticides have been and are being used widely to control wireworm populations. As wireworms live underground, they are difficult to detect, making it challenging for farmers to decide when control measures are really needed. This study aimed to improve wireworm monitoring by comparing two common methods: sampling larvae directly from the soil and using bait traps placed in the ground. Field experiments were carried out in northern Italy over several years (1993–1999), with soil samples collected 3 m from each bait trap. The results showed a clear, though moderate, association between the number of larvae found in soil samples and those captured by bait traps. This association enabled us to build a conversion table that will allow technicians and farmers to assess whether or not the damage threshold set for bait traps has been exceeded when using soil-sampling methods. The results also provided us with a reliable wireworm-monitoring method for estimating wireworm damage risk whatever the soil conditions are, e.g., when temperatures or vegetation cover impair the performance of bait traps, which are generally more efficient and less time-consuming than soil sampling. Consequently, farmers and advisors will be able to make better-informed decisions, reduce unnecessary insecticide use, and support more sustainable crop-protection practices that benefit both agriculture and the environment.

## 1. Introduction

*Agriotes* are a genus of click beetles whose larvae are known as “wireworms”. Nine main species of agricultural importance can be found in Europe: *Agriotes brevis* Candèze, *A. lineatus* L., *A. litigiosus* Rossi, *A. obscurus* L., *A. proximus* L., *A. rufipalpis* Brullé, *A. sordidus* Illiger, *A. sputator* L., and *A. ustulatus* Schäller *(Elateridae: Elaterinae: Agriotini*) [1]. They are among the main soil-dwelling pests in Europe and may cause significant damage to a variety of crops, such as maize, potato and sunflower [2,3]. Some of these species are also important in North America [4]. The widespread and often preventive use of insecticides, which is not always justified by the actual presence of damaging populations, can have serious ecological consequences, including biodiversity loss and broader effects on agricultural ecosystems [5,6]. Consequently, accurate monitoring of *Agriotes* populations is essential for their sustainable and targeted management, with it also helping to reduce pesticide use and adhere to the principles of Integrated Pest Management (IPM) [7].

The main approaches for monitoring *Agriotes* populations are soil-larval sampling with bait traps [8,9,10], soil samplers, i.e., taking soil cores [11], and pheromone traps [12,13]. Soil sampling has also been evaluated in North America with wireworm species belonging to other genera [14,15]. Pheromone traps are cost-effective tools that are well-suited to large-scale monitoring; they also identify dominant species and predict damage risk, particularly when used over multiple consecutive years [13]. This information results in the identification of fields where susceptible crops should not be sown, or where larval population levels should be monitored when susceptible crops will be sown there nevertheless [13]. It has been shown that wireworm monitoring with bait traps can reliably predict the risk of wireworm damage to the subsequent maize crop, and damage thresholds have been pinpointed for *Agriotes brevis*, *A. sordidus* and *A. ustulatus*: 1, 2 and 5 larvae per trap, respectively [8].

Bait-trap captures provide an indirect estimate of larval population levels, although the actual wireworm density remains unknown. Therefore, it would be interesting to assess whether an association exists between capture levels estimated by bait traps in a cultivated field and the density values obtained from soil sampling. It would be useful to establish this association for two reasons:

- Damage thresholds could be determined in terms of both the average number of wireworms/bait trap and larval density, as estimated with soil sampling; wireworm monitoring could thus be carried out successfully, even when soil conditions are unsuitable for bait traps (e.g., when soil is densely covered with cover crops or weeds, and in low temperatures);
- It would provide an insight into the wireworm’s ability to move in soil and reach carbon dioxide-emitting sources (e.g., bait traps).

Therefore, a long-term experiment was conducted to determine whether there was an association between bait-trap monitoring and soil sampling in the same cultivated fields.

## 2. Materials and Methods

This research was conducted from 1993 to 1999 at numerous sites in Veneto, north-east Italy, and Piedmont, in the north-west. The coordinates of the monitored sites with the number of larvae captured at each are reported in Table 1.

**Figure 1:**
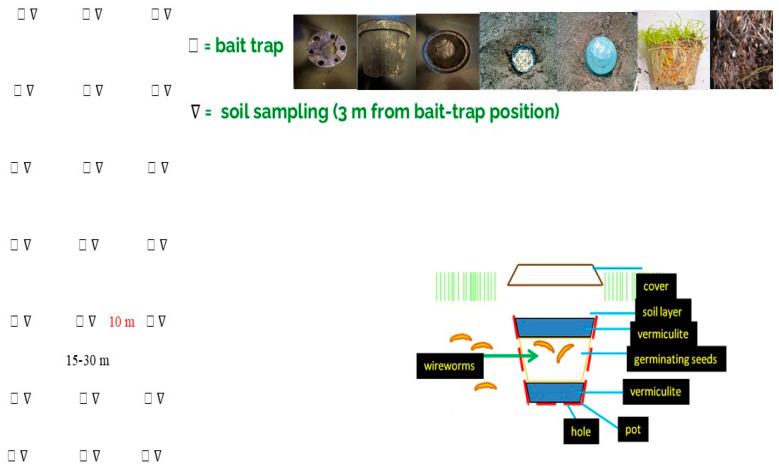
Experimental layout for comparison between soil sampling and monitoring with bait traps in the same cultivated plots of land.

Monitored fields had appreciable populations of *Agriotes brevis* Candeze, *A. sordidus* Illiger and *A. ustulatus* Schaller, being selected on the basis of previous sampling activities and the presence of agronomic risk factors [16]. Within each field, between 12 and 48 sampling points were laid out following a 10 × 15–30 m grid (Figure 1). A bait trap for larvae [8,9] was installed at every point, and a soil core was collected approximately 3 m from the trap’s location. Each trap comprised a black plastic pot (commonly used in nurseries); the pot was 10 cm in diameter at the top, 7 cm at the bottom, and 7.5 cm high. Six holes were equally distanced around the pot bottom; the hole sizes alternated between a diameter of 0.7 cm and 0.9 cm. The pots were filled with vermiculite, 30 mL of dry wheat seeds, and 30 mL of dry maize seeds; they were then moistened before being placed into the soil 4–5 cm below the surface, after which they were covered with about 2 cm of soil. An 18 cm diameter plastic lid was placed 1–2 cm above the pot rim. Traps were set in bare soil in late winter or early spring, once ground temperatures at 10 cm below the surface had reached at least 7–8 °C, following an established protocol [8]. The traps remained in the soil for 10 suitable days, occasionally longer if unsuitable weather occurred during the monitoring period [8].

Soil sampling was performed using a 12 cm diameter manual soil sampler down to a depth of 30 cm after the bait traps had been retrieved. In cases where soil temperature had not yet stabilized, an additional core was taken from 30 cm to 45–50 cm in 25% of the studied fields to recover larvae that were still in deeper soil layers. To recover all live and mobile larvae, trap contents were examined manually and then placed in 26 cm Berlese funnels fitted with a 0.5 cm mesh. Samples were allowed to dry for at least 30 days, until two consecutive inspections yielded no new larvae, and all individuals that had fallen into the collecting vials were counted and identified [1]. Soil samples (cores) were processed in the same way using 28–30 cm funnels, with a longer drying period of at least 60 days. Whereas soil sampling catches any larvae present in a single core, bait traps attract larvae moving through the soil by emitting carbon dioxide, which wireworms can detect [17].

### 2.1. Statistical Analysis

In the original dataset, each soil sample was linked to its corresponding trap located 3 m away (Figure 1).

The statistical analysis was structured in successive and increasingly complex phases to reflect the distributional properties of the data and the biological processes underlying wireworm detection. Analyses addressed both qualitative (presence/absence) and quantitative (counts) relationships between larvae detected in soil samples (SOIL CORES) and larvae captured in bait traps (TRAPS). All analyses were conducted in R Studio (R Core Team, version 4.2.2), and details on model formulations, software implementations and packages are provided in the Supplementary Materials (Annexes S1–S3).

The primary aim of this analysis was not to infer a causal relationship between soil- core and trap captures, but to assess the degree of association and functional interchangeability between the two monitoring methods. Soil-core sampling provides a direct and quantitative estimate of larval density per unit area, whereas bait traps yield an indirect estimate influenced by larval mobility and attraction to the bait. Statistical models were therefore used to evaluate whether trap data reflected soil-based larval abundance and to what extent the two methods could be considered substitutable.

#### 2.1.1. Exploratory and Descriptive Analyses

Initial exploratory analyses focused on the joint distribution of observations obtained with the two sampling methods. Observations were classified into four categories (absence in both methods, presence in traps only, presence in soil cores only, and concordant presence) and summarized by species and for the entire dataset. Summary statistics (including zero frequency, central tendency, dispersion, skewness, kurtosis, and inequality indices) were computed to characterize data structure and heterogeneity. In particular, the Gini coefficient was computed as an additional measure of inequality in larval counts across samples.

Each soil-sample/trap pair (SOIL CORES vs. TRAPS) was treated as a spatially associated observation. For each insect species, paired observations were classified into four concordance categories: absence in both methods; absence in soil but presence in traps; presence in soil but absence in traps; and presence in both methods. To assess whether the observed agreement depended on the 3 m spatial pairing between soil cores and traps, a permutation procedure was applied. Specifically, soil-core and trap observations were randomly re-paired within the same sampling unit (field), thereby breaking the original 3 m pairing while preserving the marginal detection frequencies of each method. If the observed agreement did not differ from the distribution obtained from the permuted datasets, this indicated that agreement was not specific to the 3 m pairing and may have arisen at the site level.

#### 2.1.2. Presence/Absence Association Analyses

For each species and for the pooled dataset, 2 × 2 contingency tables were constructed to evaluate the association between larval presence/absence in soil samples (SOIL CORES) and traps (TRAPS). Pearson’s Chi-squared test and Fisher’s Exact test were used to assess independence between methods, and the Phi coefficient was calculated as a measure of association strength.

To test explicitly whether the observed agreement between SOIL CORES and TRAPS depended on the original 3 m spatial pairing (i.e., whether concordance was stronger than expected under random spatial association), a permutation test was implemented. For each species, the observed Chi-squared statistic was first computed from the contingency table based on the original spatially paired observations.

Subsequently, the spatial pairing between soil cores and traps was randomly permuted within each field, thereby breaking the original 3 m pairing while preserving the site-level structure and the marginal detection frequencies of each method. For each randomized dataset, a new Chi-squared statistic was computed. This procedure was repeated 600,000 times to generate a null distribution of Chi-squared values representing the expectation under spatially random pairing.

A permutation *p*-value was then estimated as the proportion of randomized Chi-squared values equal to or greater than the observed Chi-squared statistic. This procedure directly tested whether the observed concordance between soil cores and traps at the 3 m scale exceeded what would be expected by chance given the overall detection frequencies and site-level aggregation patterns. This permutation framework avoided pseudo-replication arising from large, bootstrapped sample sizes and allowed formal inference on spatial concordance at the fine scale.

#### 2.1.3. Non-Parametric Correlation Analyses

As larval-count data were highly skewed and zero-inflated, traditional parametric correlation measures were deemed inappropriate. The analytical approach was structured in progressive phases, starting with simple non-parametric correlation analyses and advancing to more complex statistical models accounting for zero inflation and overdispersion. Non-parametric techniques were applied to explore non-linear relationships and detect monotonic associations without constraints of linearity or specific distributional forms. These methods proved insightful in uncovering potential patterns that might have remained hidden had only traditional parametric methods been used. Spearman’s rank correlation coefficient (ρ) and Kendall’s tau (τ) were computed to evaluate monotonic associations between larval counts in soil cores and trap captures for each species. Kendall’s τ was considered particularly suitable given the large number of tied values resulting from frequent zeros [18]. These analyses provided an initial quantitative indication of association strength and guided subsequent modeling steps, but were not intended to fully describe the underlying relationship.

#### 2.1.4. Diagnostic Assessment of Parametric Assumptions

Prior to fitting regression-based models, the suitability of Gaussian assumptions was formally evaluated. Linear regression and ANOVA-type models were initially tested for comparison purposes. Model assumptions were assessed using the Shapiro–Wilk test for normality, the Breusch–Pagan test for heteroscedasticity, and the Durbin–Watson test for autocorrelation. These diagnostics were applied to raw TRAPS data and to log- and square-root-transformed values; the reason was that TRAPS data were used as the response variable in the regression models, whereas SOIL CORES data were included only as explanatory variables. The persistence of non-normality, heteroscedasticity and residual dependence across all transformations demonstrated that standard linear modeling frameworks were inappropriate for these data, motivating the adoption of count-based models.

#### 2.1.5. Count-Based Regression Models

From this point onward, all analyses were conducted using the original (untransformed) TRAPS and SOIL CORES data. When modeling the relationship between the number of larvae captured by bait traps (TRAPS) and the number of larvae detected in soil samples (SOIL CORES), the objective was to evaluate whether variation in trap captures could be explained by soil-based larval density estimates. Accordingly, TRAPS were treated as the response variable, while SOIL CORES were included as the main explanatory variable. Given the discrete nature of the data, Generalized Linear Models (GLMs) for counts were subsequently fitted. A Poisson GLM (GLM-Poisson) was considered as the simplest formulation, followed by Quasi-Poisson (GLM-quasi-Poisson) and Negative Binomial (NegBin) models to account for overdispersion. Overdispersion was evaluated using dispersion statistics and residual diagnostics. As preliminary analyses revealed a large excess of zero observations, models explicitly accounting for zero inflation were then fitted. These included Hurdle Negative Binomial models and Zero-Inflated Poisson (ZIP) and Zero-Inflated Negative Binomial (ZINB) models. The zero-inflated models allowed zeros to arise from two distinct processes: (i) true absence of larvae, modeled by the zero-inflation (structural-zero) component, and (ii) non-detection despite presence, modeled by the count component, which is particularly relevant in ecological sampling contexts. For comparison purposes, Generalized Least Squares (GLS) models with heteroscedastic variance structures and Linear Mixed-Effects Models (LMMs) were also fitted, although these models do not explicitly address zero inflation.

All model formulas, including link functions, predictors and response structures, are provided in full detail in the Supplementary Materials (Annexes S1–S3).

#### 2.1.6. Model Comparison and Selection

Models were specified to characterize the statistical relationship between soil and trap data and to assess alternative error structures (e.g., overdispersion and zero inflation), rather than to derive operational conversion functions between the two sampling methods. Model selection followed an information-theoretic framework to compare competing models, while accounting for both goodness-of-fit and model complexity. Model comparisons were first performed on the pooled dataset and subsequently repeated for each species to assess species-specific differences in model performance.

For likelihood-based models, Akaike’s Information Criterion (AIC) and Bayesian Information Criterion (BIC) were used, with lower values indicating stronger model support. These likelihood-based models included Poisson GLM (GLM-Poisson), Negative Binomial (NegBin), Hurdle Negative Binomial (Hurdle), Zero-Inflated Poisson (ZIP), and Zero-Inflated Negative Binomial (ZINB). These information-theoretic criteria accounted for both goodness-of-fit and model complexity and, when model performance was comparable, they favored less complex models. Differences in AIC (ΔAIC) were used to assess relative model performance.

For quasi-likelihood models, for which a true likelihood cannot be computed, model adequacy was evaluated using dispersion parameters and residual diagnostics rather than information criteria. This included the Quasi-Poisson GLM (GLM-quasi-Poisson).

The abovementioned linear-based models (LM, LMM and GLS) were also fitted for comparison purposes. While these models do not rely on likelihood-based criteria, dispersion and variance heterogeneity were accounted for either within the model framework or via residual diagnostics.

Dispersion parameters were used to assess departures from the Poisson distribution assumption, with a dispersion value of 1 indicating equidispersion and values greater than 1 indicating overdispersion.

For Linear Mixed-Effects Models (LMMs), the findings reported both marginal R^2^ (R^2^m), representing variance explained by fixed effects, and conditional R^2^ (R^2^c), representing variance explained by both fixed and random effects.

For Linear Models (LMs) and Generalized Least Squares models (GLS), variance structure was accounted for within the model framework, and dispersion was therefore not estimated explicitly.

For the zero-inflated and hurdle models, zero-inflation probability was used to estimate the proportion of structural zeros relative to zeros generated by the count process.

Although model-based predictions were used to interpret the relationship between SOIL CORES and TRAPS data, a simple ratio-based approach was ultimately adopted for the practical conversion between sampling methods, as it provided easily applicable conversion factors for operational use. The discrepancy between ratio-based conversion factors and model-based predictions was therefore explicitly acknowledged and discussed.

All actual model formulas, as implemented in R, are fully reported in the Supplementary Materials (Annexes S1–S3).

#### 2.1.7. Model Validation and Performance Assessment

The final selected models were validated by a comprehensive assessment of goodness-of-fit, evaluated by comparing observed and model-predicted values within the same dataset. No separate test set or cross-validation was applied; thus, “predictive performance” here refers to model fit on the available data rather than prediction on independent sites. Residual diagnostics included inspection of Pearson and deviance residuals, evaluation of remaining overdispersion, and assessment of fitted versus observed values. For the zero-inflated and hurdle models, observed and predicted zero frequencies were compared to evaluate the adequacy of the zero-generating component. Predictive performance was further quantified using Root Mean Square Error (RMSE), Median Absolute Deviation (MAD), and correlation between observed and model-predicted trap counts. Incidence Rate Ratios (IRR) were calculated to quantify the effect of larval abundance in soil cores on expected trap captures. IRR represented the multiplicative change in the expected number of larvae captured by bait traps for each additional larva detected in a soil core. RMSE was defined as the square root of the mean squared difference between observed and predicted values and reflected predictive accuracy with greater sensitivity to large errors, whereas MAD represented the mean of the absolute differences between observed and predicted values and provided a robust measure of average prediction error that was less influenced by extreme values.

#### 2.1.8. Estimation of Mean Abundance and Variability from Zero-Inflated Data

Larval abundance of each wireworm species (*A. brevis*, *A. sordidus*, *A. ustulatus*) was recorded using soil samples (SOIL CORES) and bait traps (TRAPS). Due to the large number of zero counts, data were modeled separately for each species and sampling method using Zero-Inflated Negative Binomial (ZINB) models. Expected mean abundance was calculated as E(Y) = (1 − π)μ, where Y was the response variable, μ was the conditional mean of the negative binomial component, and π was the probability of structural zeros. The variance was computed as Var(Y) = (1 − π)(μ + μ^2^/θ) + π(1 − π)μ^2^, where θ was the dispersion parameter; Standard Deviation (SD) was obtained as the square root of the variance. Model-based estimates were derived from predicted values and averaged across observations for each species. Trap-to-soil sample ratio was calculated as the ZINB-estimated mean from bait traps divided by the ZINB-estimated mean from soil cores, i.e., R = X/Y, with Standard Deviation approximated with the delta method (first-order Taylor expansion), assuming zero covariance between X and Y. This simplification was adopted due to the separate estimation of model parameters. Damage Threshold (1, 2 and 5 larvae per trap for *A. brevis*, *A. sordidus* and *A. ustulatus,* respectively) was divided by the trap-to-soil ratio to obtain THRESHOLD ZINB, with propagated Standard Deviation. Thresholds were then converted to per m^2^ by multiplying by 88 (a soil sample is 1/88 of a square meter). All values were reported as mean ± SD.

## 3. Results

Across all three *Agriotes* species, SOIL CORES showed a high proportion of zero counts (86–94%), whereas TRAPS exhibited substantially lower zero percentages (37–53%) (Table 2). As a consequence, mean abundances were consistently higher in TRAPS than in SOIL CORES, particularly for *A. ustulatus*, where the median abundance in TRAPS was 1, compared to 0 in SOIL CORES. Measures of variability, including Interquartile Range (IQR) and Median Absolute Deviation (MAD), were negligible in SOIL CORES, highlighting limited variation in soil samples, whereas TRAPS showed greater dispersion, consistent with their lower zero-inflation. Gini coefficients were generally high across all datasets, suggesting that individuals were concentrated in a limited number of samples. Positive skewness and high kurtosis values further indicated strongly right-skewed and leptokurtic distributions, particularly in traps, reflecting infrequent but high-abundance events.

**Figure 2:**
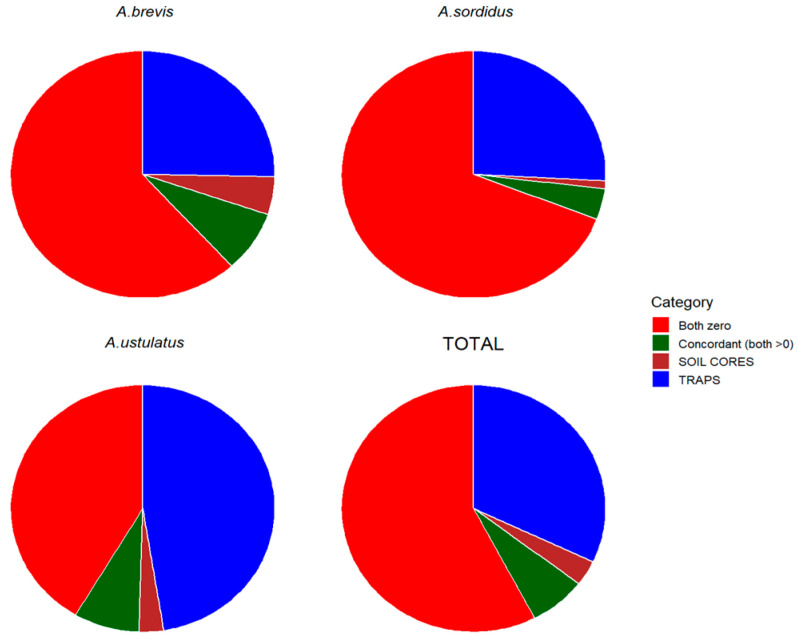
Distribution of observation categories for each species and for the overall dataset, based on the joint occurrence of larval detections in soil cores and bait traps. Colors indicate the four detection categories: Both zero: no larvae were detected by either method; Concordant: both methods detected at least one larva at the same sampling point; SOIL CORES: larvae were detected in soil cores only; TRAPS: larvae were detected in bait traps only.

In all species (Figure 2), the dominant outcome was the concurrent absence of detections in both soil cores and bait traps (red), which accounted for roughly two-thirds to three-quarters of all observations, depending on the species. This confirmed that most sampling points did not detect any larval activity with either method. Samples positive only in TRAPS (blue) constituted the second most frequent category, accounting for roughly one-quarter of observations in *A. brevis* and *A. sordidus*, and approaching 50% in *A. ustulatus*. Concordant detections (green) were relatively uncommon (below 10%) while detections restricted to SOIL CORES (brown) consistently represented the smallest proportion, usually only a small percentage of samples.

*A. ustulatus* displayed the most distinct pattern of the three species studied. Its proportion of trap-only detections was substantially higher than for *A. brevis* and *A. sordidus.* In contrast, *A. brevis* and *A. sordidus* showed more similar percentage distributions across categories.

The contingency analysis (Table 3) evaluated the agreement between the presence/absence of wireworms (*Agriotes* spp.) in the soil.

For *A. brevis*, the statistical tests (Pearson’s Chi-squared and Fisher’s Exact) yielded highly significant results (*p* < 0.001), indicating a strong and consistent association between larval presence in the soil cores and trap captures. The high proportion of concordant observations (“both absence” and “both presence”) showed that the two sampling methods were consistent and provided coherent information on the spatial distribution of *A. brevis* populations.

A similarly strong relationship was observed for *A. sordidus*, with the statistical tests again highly significant (*p* < 0.001). In contrast, *A. ustulatus* exhibited a weaker, though still statistically significant, relationship between the two methods (*p* < 0.05 for Chi-squared and Fisher’s tests).

Excluding double-zero observations (i.e., samples where larvae were absent in both methods), the proportion of concordant observations, where larvae were detected in both soil cores and bait traps, was relatively low (Table 3). Specifically, for *A. brevis*, 8.02% of observations were concordant, while 25.31% of observations corresponded to larvae detected in traps but not in soil cores, and 4.94% showed larvae in soil cores but not in traps. For *A. sordidus*, concordant observations represented 4.03% of cases, with 25.84% of samples positive in traps only, and 1.01% positive in soil cores only. *A. ustulatus* exhibited a different pattern, with 8.06% concordant, 47.46% of observations positive in traps only, and 2.99% positive in soil cores only.

The permutation test (Table 4) revealed that the observed agreement between soil cores and traps at the 3 m scale was not stronger than expected under random spatial pairing for *A. brevis* (permutation *p* = 0.146) and *A. ustulatus* (permutation *p* = 0.53), indicating that for these two species, the apparent concordance mainly reflected site-level patterns and the high frequency of joint absences rather than true spatial correspondence. In contrast, *A. sordidus* showed a weak but statistically significant fine-scale spatial association (permutation *p* = 0.0288), suggesting a limited but detectable spatial concordance.

Across species, joint absences represented the most frequent detection category, followed by discordant detections in which larvae were detected in traps but not in soil cores (Table 3). Concordant presences and soil-only detections were consistently rare. Species-specific detection patterns were evident: *A. brevis* and *A. sordidus* were characterized by high proportions of joint absences and low concordant presences, whereas *A. ustulatus* showed a markedly higher proportion of “trap-only” detections, reflecting a stronger relative responsiveness to bait traps compared with direct detection in soil cores.

To evaluate the association between the two detection methods, the joint distributions of observations referring to the presence of larvae in the soil and trap catches were analyzed.

Table 5 reports the contingency table aggregated over the entire dataset. Sites with larvae present in soil cores showed a substantially higher proportion of trap captures (78/115, 67.8%) compared with sites where larvae were present in lower numbers in soil cores (359/1004, 35.8%). This suggested that trap captures partially reflected the underlying presence of larvae in the soil, although a substantial fraction of trap positives also occurred where no larvae were detected with soil sampling.

The Chi-square test (Table 6) indicated a highly significant association between the two detection methods across all species, rejecting the hypothesis of independence. The Phi coefficient denoted a moderate association, implying that the two variables were related but not strongly correlated.

The analyses presented in Figure 2 and Table 3, Table 4, Table 5 and Table 6 were based on absence/presence data and provided an initial assessment of the consistency between the two monitoring methods. The number of individuals captured will also be examined below to further evaluate the quantitative relationship between larval density as estimated by the two methods.

Given the complex nature of the TRAPS and SOIL CORES data revealed by initial diagnostic tests, traditional parametric approaches, such as Pearson’s correlation, were deemed inappropriate due to violated distributional assumptions.

Kendall’s τ was particularly suitable for the zero-inflated datasets, being less affected by tied ranks common in observations with many identical values (zeros). The resulting non-parametric correlation coefficients (Table 7) indicated consistent positive associations between larval counts in soil cores and captures in bait traps across all *Agriotes* species, with varying strengths.

**Figure 3:**
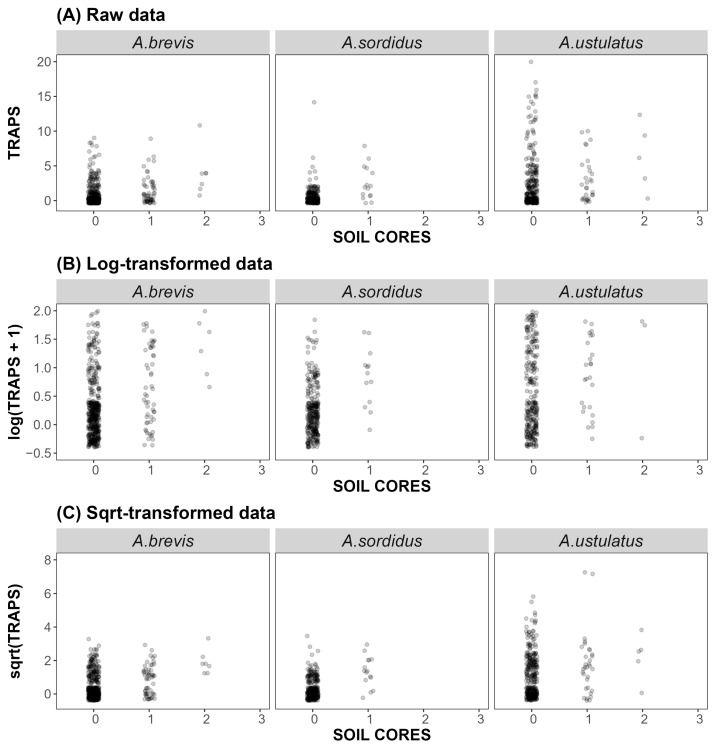
Scatter plots illustrating the relationship between the number of *Agriotes* larvae captured in bait traps (TRAPS) and the number of larvae found in soil samples (SOIL CORES) for *A. brevis*, *A. sordidus* and *A. ustulatus*. Panel (**A**) shows the raw data, Panel (**B**) the log-transformed values [log(TRAP + 1)], and Panel (**C**) the square-root-transformed values [sqrt(TRAP)]. To better visualize the high density of overlapping data points, a geometric jitter was applied.

A visual examination of the scatter plots (Figure 3), which illustrate the relationship between *Agriotes* larvae detected in soil cores and those captured in bait traps, revealed a clear methodological issue: data distributions were markedly non-normal across all larval abundance categories and for each of the three *Agriotes* species. The majority of observations were concentrated at low larval counts, with a high frequency of zero values, indicating that both soil sampling and bait traps often resulted in no detection of individuals.

Despite the data transformations (logarithmic and square-root), the overall pattern remains largely unchanged, with evident clustering near the origin and a limited range of higher values. Most data points occurred at the origin (SOIL CORES = 0, TRAPS = 0), and non-zero values were strongly right-skewed, with the majority of captures at low larvae counts and a few high trap catches.

These distributional features violated the fundamental assumptions of standard parametric statistical approaches, such as Analysis of Variance (ANOVA) and linear regression. The data showed strong deviations from normality and high zero-inflation, suggesting that parametric models may not be appropriate.

To further investigate the relationship between larval counts in soil cores and trap captures, a Generalized Linear Model (GLM) with a Gaussian error distribution was initially tested, treating soil core larval counts as the explanatory variable and trap captures as the response. However, diagnostic tests indicated that the assumptions of ANOVA-type models were not met. As shown in Table 8, Shapiro–Wilk, Breusch–Pagan and Durbin–Watson tests were applied to the TRAPS data and to their transformations (log(TRAPS + 1) and sqrt(TRAPS)), revealing significant deviations from normality (*p* < 0.001), heteroscedasticity and possible autocorrelation. These results indicated that the TRAPS data violated key assumptions of linear regression models and that standard data transformations were insufficient for addressing these issues adequately. To address these data characteristics adequately, analysis was conducted using modeling frameworks specifically designed for count data. Given the complex nature of the TRAPS data revealed by diagnostic tests, more sophisticated modeling techniques were employed. In particular, the use of Generalized Linear Models (GLMs) with non-Gaussian error distribution was explored.

Among the tested models (Table 9), the Zero-inflated Negative Binomial (ZINB) model showed the lowest AIC and BIC values (2932 and 2977, respectively), indicating the best overall fit among the candidate models. The Poisson generalized linear model exhibited strong overdispersion (dispersion = 5.07), whereas the standard Negative Binomial model adequately accounted for extra-Poisson variability (dispersion ≈ 1.10) but did not explicitly model the excess of zeros.

In the ZINB framework, zero counts were partitioned between the count component, which was modeled by a Negative Binomial distribution, and a separate zero-inflation component. The estimated zero-inflation probability was relatively low (0.098), indicating that only a small fraction of observed zeros in TRAPS were assigned to the zero-inflation process, while the majority of zeros were accommodated within the Negative Binomial count component. Importantly, the relationship between SOIL CORES and TRAPS was statistically significant (*p* = 1.20 × 10^−6^), confirming that larval abundance in soil had a measurable effect on trap captures, even when accounting for zero inflation. Marginal and conditional coefficients of determination further supported this result. SOIL CORES explained a limited proportion of variance in TRAPS (R^2^ marginal = 0.21), whereas a much larger proportion was explained when accounting for random effects and latent processes captured by the ZINB structure (R^2^ conditional = 0.80).

Compared with ZINB, alternative zero-inflated models allocated a larger fraction of zeros to the zero-inflation component (ZIP = 0.296; Hurdle = 0.557) and showed lower goodness-of-fit. Based on these results, the ZINB and ZIP models were selected for subsequent species-specific analyses of *A. brevis*, *A. sordidus* and *A. ustulatus*.

Species-specific comparisons between Zero-Inflated Poisson (ZIP) and Zero-Inflated Negative Binomial (ZINB) models are reported in Table 10.

For *A. brevis*, the Zero-Inflated Poisson (ZIP) model estimated a moderate level of zero inflation (≈0.34) with no evidence of overdispersion (dispersion = 1.00). By contrast, the Zero-Inflated Negative Binomial (ZINB) model reduced the estimated zero inflation to approximately 0.10 and accounted for mild overdispersion (dispersion = 1.22). The lower AIC (1077.88 vs. 1114.61) and BIC (1098.82 vs. 1131.36) for the ZINB model indicated a better fit to the data.

For *A. sordidus*, the ZIP model suggested a low zero-inflation probability (≈0.17) and no overdispersion. The ZINB model, however, nearly eliminated zero inflation (≈1.5 × 10^−8^) while capturing substantial overdispersion (dispersion = 1.83). The lower AIC (516.01 vs. 529.84) and BIC (534.50 vs. 544.63) indicated better model fit for the ZINB model.

In the case of *A. ustulatus*, the ZIP model indicated moderate zero inflation (≈0.30) with no overdispersion. The ZINB model again reduced zero inflation (≈0.12) and accounted for slight overdispersion (dispersion = 1.14), with considerably lower AIC (1343.53 vs. 1663.91) and BIC (1362.60 vs. 1679.17), demonstrating improved model fit.

These results suggested that larval counts across all three species were characterized by both excess zeros and overdispersion. The ZINB models consistently provided a more accurate representation of the data by simultaneously accounting for these two features.

Overall, *Agriotes ustulatus* showed the highest trap counts and the strongest deviations from proportionality when compared with the other species. In the ZINB models, *Agriotes brevis* displayed a moderate increase in trap captures with soil larvae (dispersion = 1.22; zero-inflation ≈ 0.10). In *Agriotes sordidus*, trap-capture variability was primarily due to overdispersion (dispersion = 1.83), with near-zero zero-inflation.

In all species-specific ZINB models, the expected number of larvae captured in traps (μ_TRAPS_) was modeled as a function of larval density in soil samples (SOIL_CORES) using a log link:

*A. ustulatus*: log(μ_TRAPS_) = 1.212 + 0.567·SOIL_CORES;

*A. brevis*: log(μ_TRAPS_) = −0.200 + 0.799·SOIL_CORES;

*A. sordidus*: log(μ_TRAPS_) = −0.801 + 1.757·SOIL_CORES.

**Figure 4:**
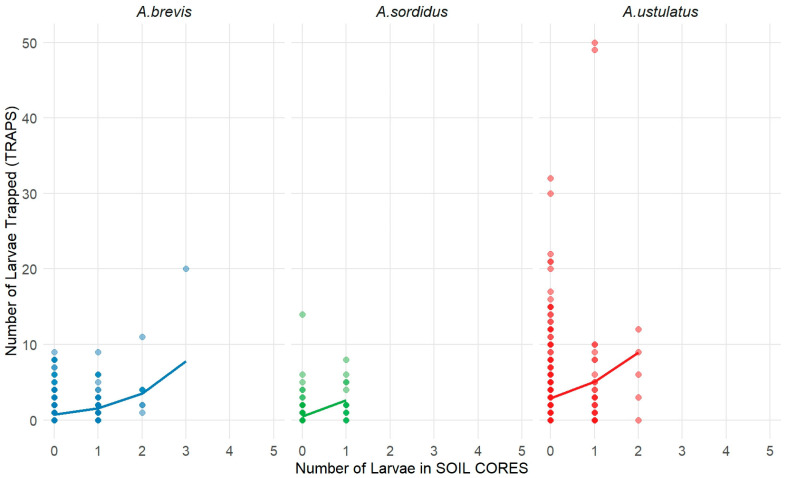
Zero-Inflated Negative Binomial (ZINB) model describing the relationship between larval counts in soil samples (SOIL CORES; x-axis) and larval captures in bait traps (TRAPS; y-axis) for *A. ustulatus*, *A. brevis* and *A. sordidus*. Points represent observed values. Colored fitted lines show the ZINB-modeled predicted mean of trap counts conditional on soil core larval density, which is plotted separately for each species.

Trap catches exhibited a positive but highly heterogeneous relationship with the number of larvae detected in soil cores. The fitted ZINB curves showed increasing expected trap captures with higher larval densities, yet the magnitude and variability of this response differed substantially among species (Table 10, Figure 4).

For *A. ustulatus*, trap captures were markedly higher and more dispersed than for the other species. Maximum trap values exceeded 40 individuals, and even when soil cores contained 0–1 larvae, generating a vertical spread of more than one order of magnitude at the lowest soil densities. Quantitatively, the predicted trap mean increased from about 6–8 individuals when 1 larva was detected in soil to more than 20 individuals for 3–4 larvae. These values should be interpreted as model-based expected trap counts rather than as direct conversion factors between soil cores and traps, and they highlight the strong non-linearity and variability of the soil/trap relationship for this species. These values confirmed that *A. ustulatus* was by far the species with the strongest and least proportional trap response.

For *A. brevis*, trap catches rarely exceeded 8–10 individuals, and the ZINB curve rose more proportionally with soil counts. The predicted mean trap catches increased from approximately 0.5–1.0 individuals at the lowest soil counts to about 2–3 individuals when soil cores contained 3–4 larvae, illustrating a moderate but consistent relationship.

For *A. sordidus*, trap counts were generally low, with most observations below 5 individuals and a very shallow response curve. Quantitatively, predicted trap means increased only modestly, from 0.5–1.0 individuals at low soil densities to 2–3 individuals at the highest observed larval counts.

Although *A. ustulatus* had the highest absolute trap counts, *A. sordidus* showed a stronger relative increase per additional soil larva. This explained why absolute trap counts were higher for *A. ustulatus*, while the proportional response to increasing larval density was greater for *A. sordidus* (Table 11). Larval abundance in soil cores was positively associated with larval captures in bait traps for all three species, as revealed by the Zero-Inflated Negative Binomial models (Table 11). The IRR indicated that each additional larva in soil cores increased expected trap captures by 76%, 122% and 479% for *A. ustulatus*, *A. brevis* and *A. sordidus,* respectively (Table 11). Model fit, assessed as the Pearson correlation between predicted and observed trap captures, was low for *A. ustulatus* (r = 0.148), and moderate for both *A. brevis* (r = 0.472) and *A. sordidus* (r = 0.355). RMSE and MAD values further illustrated model performance (Table 11): lower values for *A. brevis* and *A. sordidus* (RMSE: 1.68, 1.24; MAD: 1.10, 0.72) indicated more accurate predictions, whereas higher values for *A. ustulatus* (RMSE: 5.82; MAD: 3.49) reflected greater variability and less precise predictive ability. These results confirmed that larval counts in soil cores were a significant predictor of trap captures, with species-specific differences in effect strength. However, the large residual variability and the non-linear, zero-inflated structure of the relationship, particularly for *A. ustulatus*, indicated that model-based predictions should be interpreted cautiously and should not be used as direct conversion tools between soil core and trap counts.

## 4. Discussion

Across all species, soil cores yielded a high proportion of zero counts, reflecting the frequent absence of individuals in the sampled soil. In contrast, traps exhibited substantially lower zero counts, reflecting higher detection efficiency for actively moving wireworms. Accordingly, both mean and median larval abundances were consistently greater in traps than in soil cores, particularly for *A. ustulatus*, where the median trap count was 1 versus 0 in soil cores (Table 2, Table 3 and Table 4, Figure 2). Measures of variability showed that individuals were concentrated in a limited number of samples, indicating that wireworms of the studied species have primarily an aggregated distribution, while in a previous study [11], this distribution occurred, but was not prevalent.

*A. ustulatus* displayed the highest proportion of trap-only detections, indicating a stronger behavioral response to the food-based attractant or a higher movement capacity that increased the probability of an individual wireworm finding the bait trap. Although a general correspondence existed between larvae in soil cores and larvae captured by traps, the association was more variable or inconsistent. The strong correspondence observed for *A. brevis* and *A. sordidus* supported the reliability of bait traps as indicators of underground larval populations.

Permutation analyses indicated that, for *A. brevis* and *A. ustulatus*, the apparent concordance mainly reflected joint absences and differences between sampling sites in overall larval densities, rather than true fine-scale spatial correspondence. In contrast, *A. sordidus* showed a slight but detectable fine-scale concordance between soil core and trap detections, suggesting that, for this species, the two sampling methods tended to detect some of the same individual larvae rather than entirely independent portions of the population.

Our findings demonstrated that the relationship between the number of *Agriotes* larvae in soil cores and captures in bait traps was statistically significant but only moderate in strength, indicating that both methods reflected the same wireworm distribution in the soil. Significant Chi-square and Phi statistics confirmed an overall correspondence between methods; however, this concordance was largely driven by the simultaneous absence of insects in both traps and soil samples (double-zero observations). When these double-zero cases were excluded, discrepancies between the two methods became more evident, showing that positive detections were not always shared. From an applied perspective, this meant that when larvae were present in the soil at a site, bait traps had a higher probability of capturing at least one individual, even if the absolute counts differed. Thus, while traps and soil cores did not always yield identical counts, bait traps remained a more powerful tool for detecting the presence of larvae and identifying infested sites, particularly at the scale of individual sampling locations.

The non-parametric correlation analyses supported the previous observation that although both bait traps and soil cores generally had the capacity to detect larval presence, they did not always share positive detections. Both Spearman’s ρ and Kendall’s τ revealed that the two methods shared positive but modest associations between larval densities. The correlations were strongest for *A. brevis* and *A. sordidus*, whereas *A. ustulatus* exhibited a markedly weaker relationship.

Although Zero-Inflated Negative Binomial (ZINB) models are mathematically structured to differentiate between structural absences and sampling zeros, we did not conclude that the majority of zero observations represented sampling zeros in reality. Instead, we state herein that the ZINB model provided the best statistical fit for the data, accommodating both overdispersion and the high frequency of zeros. Given that the surveys were performed in agricultural areas historically prone to *Agriotes*, true ecological absences were expected to be rare. However, local heterogeneity and areas without larvae may also generate zeros, including structural zeros, which the model accounted for statistically. In this context, the ZINB framework was employed as a robust statistical tool to capture the observed data structure, rather than to infer the ecological nature of zeros.

Model comparison identified the Zero-Inflated Negative Binomial (ZINB) model as the most appropriate framework for describing the relationship between larval counts and trap captures. ZINB models consistently produced the lowest AIC and BIC values, effectively accommodating both excess zeros and overdispersion, and yielding the highest conditional R^2^. Model comparison identified the Zero-Inflated Negative Binomial (ZINB) model as the most appropriate framework for describing the relationship between larval counts and trap captures. These results indicated that the ZINB model provided the best statistical fit for the data. Despite the relatively low estimated share of structural zeros (≈10%), the statistical superiority of the ZINB model in our study was consistent with the behavior of ecological count data characterized by high overdispersion. According to Zuur et al. [19], even a small excess of zeros can lead to a standard Negative Binomial model failing to describe the variance-to-mean relationship adequately. Biologically, it is known that larval populations of *Agriotes* spp. can be patchily distributed (clumped) in soil [11,14,20], a trait that inherently generates an ‘excess’ of zeros in small-volume samples such as soil cores. This probably originates from the behavior of female click beetles, which lay clusters of several eggs, causing an initial concentration of small larvae [21,22]. In addition, female movements to suitable egg-laying sites are triggered by plant volatiles and/or pheromones [23,24,25,26,27], driving a further egg/larval concentration for all of the three species studied. This explanation is supported by the results of Furlan and Burgio [11], who demonstrated that the distribution of young/small *A. ustulatus* larvae was more aggregated than the spatial distribution for old last-instar larvae, and Doane [14], who demonstrated that the small larvae of *Ctenicera destructor* were much more aggregated than the large ones. While growing, the larvae move from oviposition sites and disperse, reducing population aggregation. These considerations indicated that, under random sampling with soil cores and bait traps, the probability of obtaining zero captures was high, whereas the likelihood of detecting additional wireworms in the vicinity of a soil core or bait trap where larvae were recorded was higher than in areas with zero-larvae samples. While the majority of these zeros were likely to be ‘sampling zeros’ (false negatives), the ZINB framework provided the necessary flexibility to separate the Bernoulli process (modeling the probability of presence) from the count process (modeling the density). As argued by Martin et al. [28], ignoring even a small zero-inflation component can lead to significant bias in the estimation of model parameters. Therefore, the ZINB model was selected not because structural absences were the dominant feature of the fields, but because it provided the most robust filter for the stochastic noise of the sampling process, leading to more reliable conversion thresholds for IPM application [29,30]. In contrast, Zero-Inflated Poisson models tended to overestimate the zero component, while simpler Poisson or linear models failed to capture the observed data dispersion [29,31]. It might also indicate the need to consider other factors, or to further investigate the processes that generated many zeros in the dataset [32,33].

This pattern was further supported by the contingency tables (Table 3 and Table 5), which showed the predominance of zero-zero observations alongside a higher proportion of positive trap captures when larvae were present in soil cores. Moderate but significant non-parametric correlations (Table 7) and the scatter plots (Figure 3) also reflected this tendency, with a clustering of zeros and a tendency toward co-occurrence of positive detections, consistent across the three *Agriotes* species.

The capture potential for bait traps was significantly higher than for soil sampling, as shown in Table 12 (Ratio Traps/Cores), thus the probability of finding zero-capture points on the same soil plot was lower when bait traps were used. This was due to the carbon dioxide emitted by the germinating seeds and seedlings in the bait traps that attracted the larvae over some days, while only the larvae present in the core at that precise moment could be caught. Monitoring with bait traps simply exploited the natural mechanisms that enabled larvae to find seeds and seedlings to feed on. The traps tended to capture higher numbers of larvae even when larval densities in the soil were low, thereby weakening the statistical relationship between the two detection methods.

Since the relationship between the number of *Agriotes* larvae in soil cores and captures in bait traps was statistically significant for all three species according to the ZINB models, and supported by the permutation test at the 3 m scale for *A. sordidus*, we converted the established wireworm damage thresholds in maize based on the average number of wireworms per bait trap [8] into the corresponding average number of wireworms per soil sample and the relative density in the soil (Table 12). These conversions were therefore grounded in significant model results, providing practical guidance for estimating soil larval abundance from trap data. The thresholds set with bait traps for maize resulted in soil sampling thresholds between about 15 and 17 larvae/m^2^. No peer-reviewed papers have reported thresholds based on soil sampling from fields, although some “grey” journals have reported indications without any clear description of the methods applied. According to Le Nail [34], wireworm populations can cause conspicuous plant damage to maize, sugar beet and tobacco when they exceed the threshold of 20 larvae/m^2^. The thresholds for potato and cereal crops were established at 30 larvae/m^2^. Said thresholds did not refer to one species, but to wireworms generically; however, Le Nail [34] did describe *A. lineatus*, *A. obscurus* and *A. sputator* as the main species in the area studied. Although Suss [35] provided a description of the methods adopted, it was incomplete and not very detailed. Details, however, included the sampling of 12 cm diameter cores, as in the present study, 15 cm deep, with 10 samples taken diagonally per plot, or every 10 m, according to indications by Khinkin [36]. No details were provided about the larvae-extraction method, although it appears to be manual, or how damage to plants and yield was assessed. The author reported that up to 12–15 larvae per square meter of wireworms did not lead to appreciable plant losses or a decrease in yield. However, with populations between 28 and 46 larvae per square meter, high damage was observed. The author referred generically to wireworm larvae without having determined the species being researched. Subsequent research [37] established that *A. sordidus* and *A. brevis* were the prevalent wireworms in corn rotations in the Po Valley (*A. ustulatus* was also prevalent in the eastern part); research by Suss [35] was mainly conducted here, as well.

More than one century ago (1914–1922), Roebuck [38] took from five to twelve soil samples per field (mainly plowed-up pastures). The size of the soil samples was 0.052 m^2^ (about 23 × 23 cm) and 30 cm in depth. Soil was crumbled and sifted to recover the wireworms. Although no larval identification was given, and it is possible that wireworm density could have been overestimated, the damage thresholds suggested are close to the numbers found in the present study. Roebuck also found that wireworm damage to all the crops (turnip, pea, bean, swede, cabbage, kale, mangold, cereal, linseed, potato) was negligible, being below 12.4 larvae/m^2^. Low wireworm damage to most of the crops was observed from 12.4 to 24.7 larvae/m^2^, while from 24.7 to 49.4 larvae/m^2^ was tolerably safe for cereals, broadcast crops and established plants. Beans, as later papers confirmed [39], were a tolerably safe crop where wireworms were numerous. Regarding cereals, oats proved to be more tolerant to wireworms than winter wheat and barley, which were the most susceptible. Surprisingly and encouragingly, the threshold values found in old “grey” literature were close to the thresholds for maize found in our study, which were between about 14 and 17 wireworms/m^2^ (Table 12).

Our discoveries have enabled us to achieve the first goal of this research, i.e., to estimate a bait-trap catch value from soil sampling so that a risk assessment based on soil sampling catches can be performed, and vice versa.

For research and practical purposes, we have also established a minimum number of soil samples that should be taken to ensure a reliable estimate of wireworm populations. When looking for population levels around the thresholds (Table 12), Furlan and Burgio [11] found that 16 (*A. brevis*) and 13 (*A. ustulatus*) were the minimum number of soil cores to be taken for an error of approximately 25%. For higher precision, more than 40 soil cores were needed.

As for wireworms’ ability to move through soil and reach carbon dioxide-emitting sources, our results highlighted that the potential of bait traps (with germinating seeds) to attract and catch wireworms was 5 to 25 times higher than the potential of soil sampling. Soil cores give an instant snapshot of larval presence, while bait traps estimate how many larvae in the surrounding soil can reach the carbon-dioxide source in 10 days: 1/15 of larvae in a square meter for *A. brevis*; 1/10 for *A. sordidus*; and 1/3, i.e., 5 out of 17 (Table 12), for *A. ustulatus*. These figures meant that, in theory, approximately 30% of *A. ustulatus* larvae in the 55 cm around the trap, or all the larvae in the 30 cm around the trap (i.e., 5 wireworms), could be captured. Since only some of the larvae were in the feeding phase [21,22], this meant that a significant proportion of the active larvae in a 55 cm radius may enter the trap. Some larvae from greater distances may enter the trap by chance.

## 5. Conclusions

This long-term study shows that a monitoring method to assess wireworm population levels is always available. Although developed for *Agriotes* spp., the statistical framework presented here, including permutation tests, contingency analyses, non-parametric correlations and Zero-Inflated Negative Binomial (ZINB) models, is not species-specific and may be adapted to other insect pests and agroecosystems. The permutation analysis revealed that apparent agreement between soil cores and traps was largely driven by joint absences and site-level patterns for *A. brevis* and *A. ustulatus*, whereas a weak but significant fine-scale concordance was detected for *A. sordidus*. Non-parametric correlation analyses and ZINB modeling further clarified that larval abundance in soil cores was positively associated with trap captures across all species, although species-specific patterns differ, with *A. ustulatus* showing the highest absolute trap counts and *A. sordidus* the strongest proportional response per additional soil larva. The application of these statistical approaches could support a more extensive interpretation of monitoring data in situations characterized by low detection rates and heterogeneous field conditions.

As for the practical implications of the results, a range of IPM packages can be implemented once the risk of wireworm damage has been assessed with agronomic factor evaluation [16,40] and adult pheromone traps [13]; the most suitable wireworm soil monitoring method has been identified to determine whether a wireworm population exceeds the threshold. These packages comprise tolerant crops and genotypes within a crop [41], e.g., bioactive cover crops [42,43], entomopathogens [44] and naturally derived ingredients [45], as well as other agronomic strategies and tools [46]. Overall, integrating multiple analyses provides a robust, evidence-based approach to interpreting monitoring results and supports decision-making for effective IPM strategies.

## Figures and Tables

**Table 1:** Coordinates of the monitored sites in Veneto and Piedmont with the number of monitoring points and larvae captured at each site.

REGION	PROVINCE	SITE	COORDINATES	COORDINATES	SAMPLING POINTS	SOIL SAMPLING			BAIT TRAPS		
			(N)	(E)	No.	*A. brevis*	*A. sordidus*	*A. ustulatus*	*A. brevis*	*A. sordidus*	*A. ustulatus*
PIEDMONT	TURIN	Druento	45.1520846°	7.555505°	**30**	14	0	0	78	0	0
PIEDMONT	TURIN	Poirino	44.896864°	7.827563°	**32**	14	0	0	58	0	0
PIEDMONT	TURIN	Lombriasco	44.844388°	7.637637°	**84**	8	0	0	67	0	0
PIEDMONT	TURIN	Pertusio	45.345874°	7.645953°	**34**	7	0	0	28	0	0
PIEDMONT	TURIN	Agliè	45.367628°	7.798936°	**36**	3	0	0	32	0	0
PIEDMONT	TURIN	Busano	45.325227°	7.663184°	**21**	9	0	0	27	0	0
VENETO	TREVISO	Mogliano Veneto	45.581074°	12.311373°	**15**	0	1	0	0	6	0
VENETO	TREVISO	Mogliano Veneto	45.578401°	12.316438°	**15**	1	0	0	3	0	0
VENETO	TREVISO	Mogliano Veneto	45.578948°	12.319900°	**45**	0	0	7	0	0	100
VENETO	TREVISO	Cessalto	45.698889°	12.614562°	**41**	3	0	9	8	0	0
VENETO	TREVISO	Cessalto	45.698889°	12.614562°	**70**	0	0	9	0	0	143
VENETO	VENICE	Meolo	45.621389°	12.459444°	**20**	1	0	0	6	0	0
VENETO	VENICE	Musile di Piave	45.599336°	12.555930°	**84**	0	0	7	0	0	70
VENETO	VENICE	San Donà di Piave	45.641279°	12.589317°	**19**	0	0	0	1	3	0
VENETO	VENICE	San Donà di Piave	45.648662°	12.594133°	**92**	0	0	10	0	0	331
VENETO	VENICE	San Donà di Piave	45.635663°	12.599302°	**44**	0	0	0	2	0	0
VENETO	VENICE	San Donà di Piave	45.663357°	12.604755°	**35**	1	0	0	0	0	0
VENETO	VENICE	Eraclea	45.606747°	12.643118°	**45**	0	0	4	0	0	75
VENETO	VENICE	Eraclea	45.581330°	12.702350°	**185**	29	9	0	109	27	0
VENETO	VENICE	Eraclea	45.582985°	12.707132°	**61**	6	1	0	13	10	0
VENETO	VENICE	Eraclea	45.587777°	12.779563°	**13**	0	0	1	0	0	27
VENETO	VENICE	Eraclea	45.587950°	12.782453°	**21**	0	0	7	0	0	103
VENETO	VENICE	Eraclea	45.583169°	12.808853°	**44**	0	0	5	0	0	230
VENETO	VENICE	Caorle	45.627445°	12.938679°	**63**	0	2	0	0	11	0
VENETO	VENICE	Caorle	45.639238°	12.961548°	**82**	0	13	0	0	109	0
				**TOTAL**	**1231**	**96**	**26**	**59**	**432**	**166**	**1079**

**Table 2:** Summary statistics of wireworm abundance for *Agriotes brevis*, *A. sordidus* and *A. ustulatus* obtained using soil samples (SOIL CORES) and bait traps (TRAPS). TOTAL (n), indicates the total number of soil cores taken or traps deployed; VALID (n), the number of observations without missing values; Zero Count, the number of samples with no wireworms detected; Zero Perc, the percentage of zero observations; Mean, mean number of wireworms; Median, median number of wireworms; Max, maximum number of wireworms; SD, Standard Deviation; MAD Median Absolute Deviation; IQR, Interquartile Range; Gini, Gini coefficient, a measure of inequality indicating the concentration of individuals across samples (ranges theoretically from 0, perfect uniformity, to 1, maximum inequality); Skewness, measure of asymmetry in a frequency distribution, indicating whether observations are more concentrated on the left side (negative skew) or on the right side (positive skew) of the distribution; Kurtosis, measure of the “tailedness” of a frequency distribution, indicating how strongly observations were concentrated around the mean and in the tails compared to a normal distribution (in a normal distribution, kurtosis equals 3).

Parameter	*A. brevis*		*A. sordidus*		*A. ustulatus*	
	*SOIL CORES*	*TRAPS*	*SOIL CORES*	*TRAPS*	*SOIL CORES*	*TRAPS*
***TOTAL (n)***	638	638	396	396	414	414
***VALID (n)***	629	495	396	298	406	343
***Zero Count***	547	332	372	209	363	153
***Zero Perc***	86.96	67.07	93.94	70.13	89.41	44.61
***Mean***	0.15	0.87	0.07	0.56	0.12	3.15
***Median***	0	0	0	0	0	1
***Max***	3	20	2	14	3	50
***SD***	0.425	1.850	0.268	1.328	0.384	5.848
***MAD***	0	0	0	0	0	1
***IQR***	0	1	0	1	0	4
***Gini***	0.89	0.8	0.95	0.81	0.91	0.73
***Skewness***	3.11	4.06	4.26	5.18	3.52	4.17
***Kurtosis***	10.88	27	19.04	39.4	14.2	24.77

**Table 3:** Contingency tables for *A. brevis*, *A. sordidus* and *A. ustulatus*, showing the relationship between larvae occurrence in soil samples (rows) and larvae captures in traps (columns). Each cell reports the number of observations, with percentages in parentheses. Row totals represent the overall proportion of larval absence or presence in SOIL CORES, while column totals indicate the overall proportion of trap detections or non-detections for each species. Below each contingency table, the results of Pearson’s Chi-squared test and Fisher’s Exact Test for Count Data are reported. Asterisks indicate statistical significance (* *p* < 0.05; *** *p* < 0.001).

NUMBER (PERCENTAGE)		*A. brevis*			*A. sordidus*			*A. ustulatus*		
		TRAPS			TRAPS			TRAPS		
		Absence	Presence	Total	Absence	Presence	Total	Absence	Presence	Total
SOIL CORES	Absence	300 (62%)	123 (25%)	423 (87%)	206 (69%)	77 (26%)	283 (95%)	139 (41%)	159 (47%)	298 (89%)
	Presence	24 (5%)	39 (8%)	63 (13%)	3 (1%)	12 (4%)	15 (5%)	10 (3%)	27 (8%)	37 (11%)
	Total	324 (67%)	162 (33%)	486 (100%)	209 (70%)	89 (30%)	298 (100%)	149 (44%)	186 (56%)	335 (100%)
Pearson’s Chi-squared test		*p*-value = 2.516 × 10^−7^		***	*p*-value = 1.34 × 10^−5^		***	*p*-value = 0.02353		*
Fisher’s Exact Test for Count Data		*p*-value = 6.534 × 10^−7^		***	*p*-value = 5.596 × 10^−5^		***	*p*-value = 0.03416		*

**Table 4:** Results of the permutation test of spatial association at 3 m between detections in soil cores and traps for each species. The observed χ^2^ statistic is reported along with the permutation-based *p*-value.

Species	Chi^2^ (Observed)	Permutation *p*
*A. brevis*	25.10	0.146
*A. sordidus*	16.50	0.0288
*A. ustulatus*	4.37	0.53

**Table 5:** Cumulative distribution of absence-presence observations from soil cores and trap captures across the full dataset (three species). Numbers in parentheses indicate the proportion of observations relative to the total number of observations.

LARVAE		TRAPS		
		Absence	Presence	Total
**SOIL CORES**	Absence	645 (57.6%)	359 (32.1%)	1004 (89.7%)
	Presence	37 (3.3%)	78 (7.0%)	115 (10.3%)
	Total	682 (60.9%)	437 (39.1%)	1119 (100.0%)

**Table 6:** Results of the χ^2^ test of independence and the Phi coefficient measuring the strength of association between larval presence in soil cores and trap captures. The results refer to the pooled data across all species. NA: not applicable, as no *p*-value is associated with the Phi coefficient.

Test/Index	Value	*p*-Value
Chi-square	χ^2^ = 65.2	<0.001
Phi	0.36	NA

**Table 7:** Non-parametric correlation coefficients (Spearman’s ρ and Kendall’s τ) between *Agriotes* larvae found in soil samples and captured in bait traps.

Insect Species	N (Cases)	ρ (Spearman)	*p*-Value	τ (Kendall)	*p*-Value
*A. brevis*	486	0.252	1.671 × 10^−8^	0.237	2.335 × 10^−8^
*A. sordidus*	298	0.300	1.295 × 10^−7^	0.288	2.334 × 10^−7^
*A. ustulatus*	335	0.142	9.138 × 10^−3^	0.126	9.096 × 10^−3^

**Table 8:** Results of diagnostic tests for normality, heteroscedasticity and autocorrelation on TRAPS data and their transformations.

Test	Purpose	TRAP	Log(TRAP + 1)	Sqrt(TRAP)	Test Outcome
Shapiro–Wilk	Deviation from normality	*p* < 0.001	*p* < 0.001	*p* < 0.001	Not Normal
Breusch–Pagan	Test for heteroscedasticity	*p* < 0.001	*p* < 0.001	*p* < 0.001	Heteroscedastic
Durbin–Watson	Autocorrelation in the residuals	*p* < 0.001	*p* < 0.001	*p* < 0.001	Possible autocorrelation

**Table 9:** Comparison of statistical models describing the relationship between the number of larvae captured by bait traps (TRAPS) and the number of larvae detected in soil samples (SOIL CORES). Models were calculated across all species. Reported are model type, with the abbreviation used in the text in parentheses, Akaike Information Criterion (AIC), Bayesian Information Criterion (BIC), dispersion, marginal and conditional R^2^, zero-inflation probability, and *p*-values for the SOIL_CORES effect. Models including zero-inflation explicitly partition zeros into count and zero-inflation components. Formulas are provided in the Supplementary Materials.

Model Type	AIC	BIC	Dispersion	R^2^ Marginal	R^2^ Conditional	Zero Inflation	*p* Values
Zero-inflated NegBin (ZINB)	2932	2977		0.21	0.799	0.098	1.20 × 10^−6^
Negative Binomial (NegBin)	3180	3215	1.1				8.14 × 10^−7^
Hurdle NegBin (Hurdle)	3265	3305				0.557	1.71 × 10^−2^
Zero-inflated Poisson (ZIP)	3305	3345		0.176	0.678	0.296	1.71 × 10^−14^
Poisson GLM (GLM-Poisson)	4901	4931	5.07				3.10 × 10^−84^
Generalized Least Square (GLS)	5044	5089					1.62 × 10^−11^
Linear Mixed Model (LMM)	5762	5802		0.0938	0.33		
Linear model (LM)	5954	5989					1.62 × 10^−11^
Quasi-Poisson GLM (GLM-quasi-Poisson)			5.07				1.29 × 10^−11^

**Table 10:** Model comparison for larval counts of three *Agriotes* species, in which the number of larvae captured by bait traps (TRAPS, response variable) was modeled as a function of larval density estimated from soil samples (SOIL CORES, explanatory variable). Zero-Inflated Poisson (ZIP) and Zero-Inflated Negative Binomial (ZINB) models were fitted separately for each species. Reported are the number of observations (Nobs), Akaike Information Criterion (AIC), Bayesian Information Criterion (BIC), dispersion parameter, estimated zero-inflation probability, and *p*-values for the SOIL_CORES effect. Lower AIC and BIC values indicate better model performance.

Insect	Nobs	Model	AIC	BIC	Dispersion	Zero Inflation Probability	*p* Value
*A. brevis*	486	ZIP	1114.6	1131.4	1	0.336	3.34 × 10^−12^
		ZINB	1077.9	1098.8	1.22	0.103	1.51 × 10^−3^
*A. sordidus*	298	ZIP	529.84	544.63	1	0.168	4.72 × 10^−8^
		ZINB	516.01	534.5	1.83	1.53 × 10^−8^	1.32 × 10^−5^
*A. ustulatus*	335	ZIP	1663.9	1679.2	1	0.298	1.74 × 10^−7^
		ZINB	1343.5	1362.6	1.14	0.116	0.0207

**Table 11:** Incidence Rate Ratios (IRR) for larval counts in soil cores and measures of model fit and agreement between predicted and observed values for Zero-Inflated Negative Binomial (ZINB) models fitted to three wireworm species (*Agriotes ustulatus*, *A. brevis* and *A. sordidus*). Reported statistics include Pearson’s correlation coefficient, Root Mean Square Error (RMSE) and Mean Absolute Deviation (MAD).

Wireworm Species	IRR (SOIL CORES)	Correlation (Predicted vs. Observed)	RMSE	MAD
*A. ustulatus*	1.76	0.148	5.82	3.49
*A. brevis*	2.22	0.472	1.68	1.10
*A. sordidus*	5.79	0.355	1.24	0.72

**Table 12:** Conversion of wireworm damage thresholds from bait-trap captures to soil-sampling assessments. The table reports the target species, the established IPM wireworm threshold for maize, the estimated wireworm means and standard deviations (mean ± SD) from ZINB models for both soil cores and bait traps, and the conversion ratio (R = Traps/Cores). Soil-sampling density thresholds are derived as follows: Wireworms/Core = Damage Threshold ÷ R; Wireworms/m^2^ = Wireworms/Core × 88 (sample surface area = 1/88 m^2^).

Insect Species	Damage Threshold Wireworms/Trap	Wireworms/ Soil Core	Wireworms/ Bait Trap	Ratio Traps/Cores	Threshold Wireworms/Core	Threshold Wireworms/m^2^
*A. brevis*	1	0.148 ± 0.409	0.887 ± 1.811	5.99 ± 20.56	0.167 ± 0.574	14.701 ± 50.5
*A. sordidus*	2	0.05 ± 0.224	0.557 ± 1.165	11.07 ± 54.48	0.181 ± 0.89	15.904 ± 78.295
*A. ustulatus*	5	0.125 ± 0.377	3.179 ± 5.375	25.36 ± 87.48	0.197 ± 0.68	17.352 ± 59.863

## Data Availability

The original contributions presented in this study are included in the article/Supplementary Materials. Further inquiries can be directed to the corresponding author.

## Supplementary Materials

The following supporting information can be downloaded at: https://www.mdpi.com/article/10.3390/insects17040419/s1. Annex S1: Statistical models used; Annex S2: R (version4.5.3.) Packages Used and References; Annex S3: Model Formulas. References [47,48,49,50,51,52,53,54,55,56,57,58,59,60,61,62,63,64,65,66,67] are cited in the Supplementary Materials.
