# Supplementary material for: The Association Between Soil Sampling and Bait Traps in Wireworm Monitoring: A Methodological and Statistical Approach
Source: Insects. 2026 Apr 15;17(4):419. doi: 10.3390/insects17040419 (PMC13116853; doi:10.3390/insects17040419)
Supplement: Supplementary file 1 [file insects-17-00419-s001.zip › Supplementary Materials.pdf]

insects

Supplementary Materials: The following supporting information can be downloaded at: https://www.mdpi.com/article/doi/s1. Annex S1: Statistical models used; Annex S2: R (version4.5.3.) Packages Used and References; Annex S3: Model Formulas. References [47–67] are cited in the Supplementary Materials.

Annex S1 – Statistical models used

Summary of the statistical models used to describe the relationship between larval counts (Larvae) and trap captures (TRAP) in Agriotes species, with the corresponding R packages used for model implementation.

Model type	Error distribution	Zero component	Accounts for overdispersion	Accounts for zero inflation	Main R function/package	Interpretation/ Notes
LM (Linear Model)	Gaussian (normal)	None	✗	✗	lm() – stats	Baseline model assuming continuous, normally distributed residuals.
LMM (Linear Mixed Model)	Gaussian (normal)	None	✗	✗	lmer() – lme4	Includes random effects (e.g. field, site) to account for grouped or hierarchical data.
GLM_Poisson (Poisson GLM)	Poisson	None	✗	✗	glm(family = poisson) – stats	Suitable for pure count data; assumes mean = variance.
GLM_quasiPoisson (Quasi-Poisson GLM)	Quasi-Poisson	None	✓ (via dispersion parameter)	✗	glm(family = quasipoisson) – stats	Adjusts variance without altering mean structure; does not handle excess zeros.
NegBin (Negative Binomial GLM)	Negative Binomial	None	✓	✗	glm.nb() – MASS	Models overdispersed count data (variance > mean).
Hurdle NegBin (Hurdle model)	Negative Binomial (truncated at zero)	Binary hurdle	✓	✓ (two-part model)	hurdle() – pscl	Separates zero-generation from positive-count process; assumes all zeros come from the hurdle component.
ZIP (Zero-inflated Poisson)	Poisson	Binary (logit)	✗	✓	zeroinfl(family = 'poisson') – pscl	Mixture of structural zeros and Poisson counts; may underfit overdispersed data.
ZINB (Zero-inflated Negative Binomial)	Negative Binomial	Binary (logit)	✓	✓	glmmTMB(family = nbinom2, ziformula = ~1) – glmmTMB	Simultaneously accounts for overdispersion and zero inflation; most suitable for aggregated ecological data.
GLS (Generalized Least Squares)	Gaussian (heteroscedastic)	None	✓ (via variance structure)	✗	gls() – nlme	Allows non-constant variance across groups or predictors; used for comparison with count models.

The table summarizes the statistical frameworks used to model the relationship between *Agriotes* larvae captured in bait traps (TRAP) and larvae detected in soil samples (SOIL CORES). Each model type was fitted using R packages optimized for the respective data structures: stats (R Core Team) for classical linear and generalized models; MASS for Negative Binomial regression; pscl

for hurdle and zero-inflated Poisson models; lme4 for mixed-effects models; glmmTMB for zero-inflated and mixed Negative Binomial models; and nlme for generalized least squares with heteroscedastic variance structures. This progression reflects increasing model complexity and ecological realism, from Gaussian and Poisson assumptions to models explicitly accounting for overdispersion, excess zeros and hierarchical random effects.

Annex S2 - R Packages Used and References

Library	References
**broom**	Robinson, D., Hayes, A., & Couch, S. (2023). broom: Convert Statistical Objects into Tidy Tibbles (Version 1.0.5) [Computer software]. https://cran.r-project.org/web/packages/broom/index.html
**dplyr**	Wickham, H., François, R., Henry, L., Müller, K., & Vaughan, D. (2023). dplyr: A Grammar of Data Manipulation (Version 1.1.3) [Computer software]. https://dplyr.tidyverse.org/
**tidyr**	Wickham, H., Vaughan, D., & Girlich, M. (2023). tidyr: Tidy Messy Data (Version 1.3.0) [Computer software]. https://tidyr.tidyverse.org/
**car**	Fox, J., & Weisberg, S. (2019). An R Companion to Applied Regression (3rd ed.). Sage. https://www.john-fox.ca/Companion/
**epiR**	Stevenson, M., Nunes, T., Heuer, C., Marshall, J., Sanchez, J., Thornton, R., Reiczigel, J., Robison-Cox, J., Sebastiani, P., Solymos, P., Yoshida, K., Jones, G., Pirikahu, S., Firestone, S., & Kyle, R. (2023). epiR: Tools for the Analysis of Epidemiological Data (Version 2.0.66) [Computer software]. https://CRAN.R-project.org/package=epiR
**flextable**	Gohel, D. (2023). flextable: Functions for Tabular Reporting (Version 0.9.4) [Computer software]. https://davidgohel.github.io/flextable/
**ggplot2**	Wickham, H., Chang, W., Henry, L., Pedersen, T. L., Takahashi, K., Wilke, C., Woo, K., Yutani, H., & Dunnington, D. (2023). ggplot2: Create Elegant Data Visualisations Using the Grammar of Graphics (Version 3.4.4) [Computer software]. https://ggplot2.tidyverse.org
**glmmTMB**	Brooks, M. E., Kristensen, K., van Benthem, K. J., Magnusson, A., Berg, C. W., Nielsen, A., Skaug, H. J., Mächler, M., & Bolker, B. M. (2017). glmmTMB balances speed and flexibility among packages for zero-inflated generalized linear mixed modeling. The R Journal, 9(2), 378-400. https://doi.org/10.32614/RJ-2017-066
**gridExtra**	Auguie, B. (2017). gridExtra: Miscellaneous Functions for "Grid" Graphics (Version 2.3) [Computer software]. https://CRAN.R-project.org/package=gridExtra
**lme4**	Bates, D., Mächler, M., Bolker, B., & Walker, S. (2015). Fitting linear mixed-effects models using lme4. Journal of Statistical Software, 67(1), 1-48. https://doi.org/10.18637/jss.v067.i01
**lmtest**	Zeileis, A., & Hothorn, T. (2002). Diagnostic checking in regression relationships. R News, 2(3), 7-10. https://CRAN.R-project.org/doc/Rnews/
**MASS**	Venables, W. N., & Ripley, B. D. (2002). Modern Applied Statistics with S (4th ed.). Springer. https://www.stats.ox.ac.uk/pub/MASS4/
**nlme**	Pinheiro, J., Bates, D., DebRoy, S., Sarkar, D., & R Core Team. (2023). nlme: Linear and Nonlinear Mixed Effects Models (Version 3.1-164) [Computer software]. https://CRAN.R-project.org/package=nlme
**officer**	Gohel, D. (2023). officer: Manipulation of Microsoft Word and PowerPoint Documents (Version 0.6.2) [Computer software]. https://davidgohel.github.io/officer/
**patchwork**	Pedersen, T. L. (2023). patchwork: The Composer of Plots (Version 1.1.3) [Computer software]. https://CRAN.R-project.org/package=patchwork
**performance**	Lüdecke, D., Ben-Shachar, M. S., Patil, I., Waggoner, P., & Makowski, D. (2021). performance: An R package for assessment, comparison and testing of statistical models. Journal of Open Source Software, 6(60), 3139. https://doi.org/10.21105/joss.03139
**pscl**	Jackman, S. (2023). pscl: Classes and Methods for R Developed in the Political Science Computational Laboratory (Version 1.5.9) [Computer software]. https://github.com/atahk/pscl
**psych**	Revelle, W. (2023). psych: Procedures for Psychological, Psychometric, and Personality Research (Version 2.3.9) [Computer software]. https://CRAN.R-project.org/package=psych
**purrr**	Henry, L., & Wickham, H. (2023). purrr: Functional Programming Tools (Version 1.0.2) [Computer software]. https://purrr.tidyverse.org/
**readxl**	Wickham, H., & Bryan, J. (2023). readxl: Read Excel Files (Version 1.4.3) [Computer software]. https://readxl.tidyverse.org/
**tibble**	Müller, K., & Wickham, H. (2023). tibble: Simple Data Frames (Version 3.2.1) [Computer software]. https://tibble.tidyverse.org/

Annex S3 - Model Formulas

1. Linear Model (LM):

$$Y_i = \beta_0 + \beta_1 X_i + \varepsilon_i,\ \ \varepsilon_i \sim N(0, \sigma^2)\ \ (Eq. S1)$$

Where:
- Y_i: Dependent variable (response) for observation i
- X_i: Independent variable (predictor) for observation i
- β_0: Intercept
- β_1: Regression coefficient
- ε_i: Error term for observation i, normally distributed
- σ^2: Error variance

2. Generalized Least Squares (GLS):

$$\mathrm{Y} = X\beta + \varepsilon, \varepsilon \sim N(0, \Sigma)\ \ (Eq. S2)$$

Where:
- Y: Vector of dependent variables
- X: Matrix of independent variables
- β: Vector of regression coefficients
- ε: Vector of errors
- Σ: Error covariance matrix

3. Linear Mixed Model (LMM):

$$Y_{ij} = \beta_0 + \beta_1 X_{ij} + u_j + \varepsilon_{ij},\ \ u_j \sim N(0, \sigma_u^2),\ \ \varepsilon_{ij} \sim N(0, \sigma^2)\ \ (Eq. S3)$$

Where:
- Y_{ij}: Dependent variable for observation i in group j
- X_{ij}: Independent variable for observation i in group j
- β_0: Fixed intercept
- β_1: Fixed regression coefficient
- u_j: Random effect for group j
- ε_{ij}: Error term for observation i in group j
- σ_u^2: Variance of random effects
- σ^2: Residual error variance

4. Poisson GLM:

$$\mathrm{Y_i} \sim \mathrm{Poisson}(\lambda_i),\qquad \log(\lambda_i) = \beta_0 + \beta_1 \mathrm{X_i}\ \ (\mathrm{Eq. S4})$$

Where:
- Y_i: Dependent variable (count) for observation i
- λ_i: Intensity parameter of the Poisson distribution
- β_0: Intercept
- β_1: Regression coefficient
- X_i: Independent variable for observation i

5. Quasi-Poisson GLM:

$$\mathrm{Y_i} \sim \mathrm{QP}(\lambda_i, \varphi),\qquad \log(\lambda_i) = \beta_0 + \beta_1 \mathrm{X_i}\ \ (\mathrm{Eq. S5})$$

Where:
- Y_i: Dependent variable (count) for observation i
- λ_i: Intensity parameter
- φ: Dispersion parameter
- β_0: Intercept
- β_1: Regression coefficient
- X_i: Independent variable for observation i

6. Negative Binomial GLM:

$$\mathrm{Y_i} \sim \mathrm{NegBin}(\mu_i, \theta),\qquad \log(\mu_i) = \beta_0 + \beta_1 \mathrm{X_i}\ \ (\mathrm{Eq. S6})$$

Where:
- Y_i: Dependent variable (count) for observation i
- μ_i: Mean of the negative binomial distribution
- θ: Dispersion parameter
- β_0: Intercept
- β_1: Regression coefficient
- X_i: Independent variable for observation i

7. Zero-Inflated Poisson (ZIP):

$$P(Y_i = 0) = \pi_i + (1 - \pi_i)e^{-\lambda_i},\ P(Y_i = y_i > 0) = \frac{(1 - \pi_i)\left(\lambda^{y}\ e^{-\lambda_i}\right)}{y_{i!}} \quad (\text{Eq. S7})$$

Where:
- Y_i: Dependent variable (count) for observation i
- π_i: Probability of structural zero
- λ: Intensity parameter of the Poisson distribution
- y_i: Observed value for observation i

8. Zero-Inflated Negative Binomial (ZINB):

$$P(Y_i = 0) = \pi_i + (1 - \pi_i)\left(\frac{\theta}{\mu_i + \theta}\right)^{\theta} \quad (\text{Eq. S8})$$

Where:
- Y_i: Dependent variable (count) for observation i
- π_i: Probability of structural zero
- μ_i: Mean of the negative binomial distribution
- θ: Dispersion parameter

9. Hurdle Negative Binomial:

$$P(Y_i = 0) = \pi_i,\ P(Y_i = y_i > 0) = \frac{(1 - \pi_i)\left(f_{NegBin(y_i)}\right)}{1 - f_{NegBin(0)}} \quad (\text{Eq. S9})$$

Where:
- Y_i: Dependent variable (count) for observation i
- π_i: Probability of zero
- y_i: Observed value for observation i
- f_{NegBin}: Density function of the negative binomial distribution

Disclaimer/Publisher's Note: The statements, opinions and data contained in all publications are solely those of the individual author(s) and contributor(s) and not of MDPI and/or the editor(s). MDPI and/or the editor(s) disclaim responsibility for any injury to people or property resulting from any ideas, methods, instructions or products referred to in the content.
